# Umatilla Virus in Zoo-Dwelling Cape Penguins with Hepatitis, Germany

**DOI:** 10.3201/eid3012.240498

**Published:** 2024-12

**Authors:** Monica Mirolo, Madeleine de le Roi, Katja von Dörnberg, Franziska Kaiser, Adnan Fayyad, Christina Puff, Ulrich Voigt, Ursula Siebert, Martin Ludlow, Wolfgang Baumgärtner, Albert Osterhaus

**Affiliations:** University of Veterinary Medicine, Hannover Foundation, Hannover, Germany (M. Mirolo, M. de le Roi, F. Kaiser, A. Fayyad, C. Puff, U. Voigt, U. Siebert, M. Ludlow, W. Baumgärtner, A. Osterhaus); Hannover Adventure Zoo, Hannover (K. von Dörnberg); An-Najah National University, Nablus, Palestine (A. Fayyad)

**Keywords:** Umatilla virus, viruses, zoonoses, Cape penguins, hepatitis, Germany, zoo, systemic, serology, wild birds

## Abstract

Analysis of liver tissue from a Cape penguin that died with hepatitis at a zoo in Germany revealed Umatilla virus. Testing uncovered Umatilla virus RNA in samples from 2 other deceased Cape penguins at the zoo. Our results expand knowledge of the prevalence of this virus in bird species across Germany.

Researchers first isolated Umatilla virus (UMAV) from wild birds in the United States in the 1960s, also identifying the virus in several species of *Culex* mosquitoes in Australia, Japan, China, and the United States (*1*–*5*). The low pathogenicity of UMAV has contributed to the incidental detection of the virus in wild birds. In Germany, investigators identified 3 UMAV strains (ED-I-93/19, ED-I-87/19, and ED-I-205/19) in wild bird species in 2022 (*6*). 

UMAV is a mosquitoborne arbovirus (genus *Orbivirus,* family Sedoreoviridae) with a 10-segment, double-stranded RNA genome and 7 structural proteins forming the inner and outer core. The structural RNA-dependent RNA polymerase viral protein (VP) 1 and inner-core protein VP3 are the most conserved proteins among orbiviruses. In contrast, the outer-capsid protein VP2 and, to a lesser extent, VP5 are more variable among orbivirus species and distinguish virus serotypes (*7*–*9*). The UMAV life cycle is sustained between UMAV-competent *Culicinae* mosquitoes and wild birds, the primary virus hosts. Limited studies on the UMAV sylvatic life cycle in wild birds and the respective vector species has created a paucity of knowledge regarding the diversity of susceptible hosts, the pathogenicity of the virus, and the genetic diversity of circulating strains.

## The Study

A Cape penguin (*Spheniscus demersus*) died without overt clinical signs in August 2019 at Hannover Adventure Zoo, Hannover, Germany. Histopathologic examination revealed mild-to-moderate, periportal, lymphocytic hepatitis with hepatocellular necrosis (Figure, panel A) indicative of a viral infection. Routine immunohistochemical tests for influenza virus yielded negative results (data not shown). Virus isolation from homogenized liver tissue in a chicken liver–derived (leghorn male hepatocellular [LMH]) cell line showed a cytopathic effect at 3 days postinoculation (Appendix 1 Figure 1). We extracted RNA from clarified supernatant of inoculated LMH cells and conducted next-generation sequencing according to methods previously described (*10*). We conducted bioinformatic analyses of raw next-generation sequence reads by using the CZ-ID bioinformatic pipeline (*11*), which showed 304,052 reads aligning to orbiviruses, with UMAV demonstrating the highest percent homology. We then performed nucleotide sequence analyses on the isolated UMAV strain (Umatilla virus isolate DE/Penguin/2019) ; GenBank accession nos. PP669535–44 and found it to be homologous (94%–99% identical) to ED-87-19, ED-93-19, and ED-205-19 (*6*) in all genome regions except for segment 3, where a nucleotide sequence identity ≤72% was observed with other UMAV strains from Germany (Appendix 2 Table 1). The sequence variation of the outer capsid protein has been previously observed for UMAV in Germany (*6*), and we could not rule out that it might result from a reassortment with other circulating but not yet sequenced UMAVs. 

Amino acid sequences generated from each UMAV genomic segment showed alignment with multiple orbiviruses (Appendix 2 Table 2). We constructed maximum-likelihood consensus trees with 1,000 bootstraps in accordance with the best-fit model calculated by using MEGA 11 software (*12*). Phylogenetic trees based upon amino acid sequences of UMAV proteins exhibited analogous topology (Appendix 1 Figure 2). We used the nucleotide sequence of UMAV segment 2 to design a quantitative reverse transcription PCR (qRT-PCR) and a fluorescence in situ hybridization (FISH) probe to evaluate UMAV tissue tropism. We detected UMAV RNA in hematopoietic cells, most likely macrophages, within hepatic sinusoids and in hepatocytes (Figure, panel B). We detected no marked histopathologic changes in the spleen (Figure, panel C), but macrophage-like cells in the follicle centers tested positive for UMAV by FISH (Figure, panel D). Enterocytes of the small intestine displayed mild multifocal necrosis (Figure, panel E) and were positive for UMAV by FISH (Figure, panel F); other organs were negative by nucleic acid detection methods. We noted high quantification cycle values (30–33.2) in lung, small and large intestine, and brain, and spleen, kidney, and liver showed the lowest UMAV quantification cycle values (25.5–27.2) (Table 1). In contrast to the qRT-PCR results, kidneys tested negative by FISH. Those apparently conflicting data are consistent with the lower sensitivity of FISH for formalin-fixed, paraffin-embedded (FFPE) tissues (*13*). 

**Figure:**
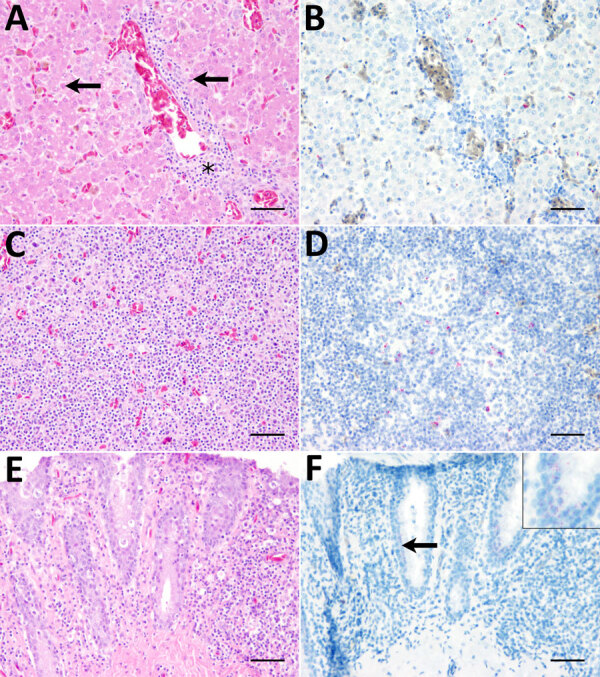
Histopathologic findings and fluorescence in situ hybridization (FISH) results in a deceased zoo-dwelling, Umatilla virus (UMAV)–infected penguin, Germany, 2019. A) Hematoxylin and eosin (H&E) stain of liver tissue shows mild, multifocal, randomly distributed, coagulative necrosis of hepatocytes (arrows) and mild-to-moderate, periportal, lymphocytic hepatitis (asterisk). B) FISH of liver tissue shows intracytoplasmically UMAV RNA-positive hepatocytes. C) H&E stain of spleen tissue shows no significant histopathologic alterations. D) FISH of spleen tissue shows multifocal cells within follicle centers, most likely macrophages, tested cytoplasmatically positive for UMAV RNA. E) H&E stain of small intestine tissue shows mild, multifocal necrosis of epithelial cells of the small intestine. F) FISH of small intestine tissue shows positive UMAV RNA signal within enterocytes. Inset: Multifocal, granular red signal within cytoplasm of enterocytes. Scale bars indicate 50 µm.

**Table 1:** Comparison of qRT-PCR and FISH results for a UMAV-infected zoo-dwelling penguin, Germany, 2019*

Sample name and source	qRT-PCR, C_q_	FISH
S849/19 liver	27.24	Positive
S849/19 kidney	26.37	Negative
S849/19 lung	30.37	Negative
S849/19 duodenum	30.04	Positive
S849/19 brain	33.22	Negative
S849/19 colon	32.69	Negative
S849/19 spleen	25.71	Positive

*C_q_, quantification cycle; FISH, fluorescence in situ hybridization; qRT-PCR, quantitative reverse transcription PCR; UMAV, Umatilla virus.

We investigated whether additional UMAV infections had occurred in other penguins from the same zoo in Germany by performing FISH on FFPE liver samples from 15 penguins that died without overt clinical signs during 2005–2021. Histologically, 9 of those penguins displayed hepatitis. UMAV RNA was detected by FISH in 2 Cape penguins, 1 dying in 2005 and 1 in 2011 (Appendix 1 Figure 3). However, we could not confirm those data by qRT-qPCR performed on RNA extracted from the same FFPE material, possibly because of poor RNA quality and fragmentation (*14*). We also screened for UMAV neutralizing antibodies in 7 serum samples from penguins and in IgY extracted from egg yolks from 9 penguins and 1 red cardinal (*Cardinalis cardinalis*) from the same zoo. We mixed serial serum sample dilutions (1:10) and IgY sample dilutions (1:20–80) and added 600 TCID_50_ (50% tissue culture infectious dose) per well of isolated UMAV, before applying the solution onto LMH cells. Positive results relied on 100% virus neutralization, determined by immunofluorescence staining of intracellular UMAV double-stranded RNA (dsRNA) by using mouse monoclonal antibody J2 (*15*). The analysis revealed no UMAV antibodies in the samples. We applied the same analysis to 94 serum samples collected from wild pheasants captured in northwestern Germany during 2011–2017. We noted an overall seroprevalence of ≤37%, the highest in 2011 (Table 2).

**Table 2:** UMAV neutralization test results for serum samples from wild-caught pheasants in northern Germany*

Year	Animal ID	Collection date	UMAV antibody titer†
2011	11005	Mar 3	1:20
	11014	Apr 4	1:20
	11019	Apr 7	1:20
	11024	Apr 7	1:40
2012	12056	Mar 21	1:40
2014	14103	Mar 11	1:40
	14108	Mar 11	>1:80

*UMAV, Umatilla virus. †The antibody titers were measured in semiquantitative scale: <1:20 serum dilution was considered as negative, and then 1:20, 1:40, 1:80, or >1:80.

## Conclusion

We isolated and molecularly characterized UMAV from the liver of a deceased Cape penguin in a zoo in Germany. This penguin had lymphocytic hepatitis and UMAV RNA in hepatocytes. qRT-PCR revealed a systemic infection with high viral loads in spleen and kidney. Phylogenetic analyses indicated that the UMAV strain involved is closely related to UMAVs isolated from wild birds in Germany. The virus caused systemic infection consistent with published findings (*6*). Two other penguins with hepatitis that died in the same zoo, 1 in 2005 and 1 in 2011, tested positive for UMAV by FISH. The use of dsRNA antibodies as an alternative virus detection method should be interpreted with caution because, as revealed in this investigation, different cell types tested UMAV-positive as determined by dsRNA in liver tissue compared with positive cell types detected by FISH. 

Serologic analysis showed evidence of UMAV transmission among free-living pheasants in northwestern Germany in 2011–2017. UMAV infections among wild birds increases the likelihood of virus transmission to additional susceptible hosts. We theorized that the carrier mosquitoes transmitted the virus from free-living wild birds to the penguins in the affected zoo.

This case study of penguins in Germany expands the collective knowledge regarding the susceptible host range for UMAV, as well as aspects of the pathogenesis and the epidemiology of UMAV infections in birds with the specific clade of virus previously identified in Germany. Our seroprevalence data indicate the need for further investigation into the susceptibility of domesticated birds, such as poultry, to UMAV infection. Controlled, in vivo infection studies of UMAV in domestic and wild bird species would be useful in better defining the virulence of this virus. Coupled with reported serologic evidence of UMAV infection in goats, horses, and donkeys in Australia (*2*), our findings suggest the need for more in-depth exploration into the potential for UMAV infection in mammal species, including humans. 

Appendix 1 Additional information for Umatilla virus in zoo-dwelling Cape penguins with hepatitis, Germany

Appendix 2 Data informing nucleotide sequence identity and phylogenetic tree construction for Umatilla virus in zoo-dwelling Cape penguins with hepatitis, Germany

## References

[1] Belaganahalli MN, Maan S, Maan NS, Tesh R, Attoui H, Mertens PP. Umatilla virus genome sequencing and phylogenetic analysis: identification of stretch lagoon orbivirus as a new member of the Umatilla virus species. PLoS One. 2011;6:e23605. 10.1371/journal.pone.0023605 21897849 PMC3163642

[2] Cowled C, Palacios G, Melville L, Weir R, Walsh S, Davis S, et al. Genetic and epidemiological characterization of Stretch Lagoon orbivirus, a novel orbivirus isolated from *Culex* and *Aedes* mosquitoes in northern Australia. J Gen Virol. 2009;90:1433–9. 10.1099/vir.0.010074-0 19282430 PMC2887561

[3] Tangudu CS, Charles J, Hurt SL, Dunphy BM, Smith RC, Bartholomay LC, et al. Skunk River virus, a novel orbivirus isolated from *Aedes trivittatus* in the United States. J Gen Virol. 2019;100:295–300. 10.1099/jgv.0.001219 30632960

[4] Ejiri H, Kuwata R, Tsuda Y, Sasaki T, Kobayashi M, Sato Y, et al. First isolation and characterization of a mosquito-borne orbivirus belonging to the species Umatilla virus in East Asia. Arch Virol. 2014;159:2675–85. 10.1007/s00705-014-2117-0 24906523

[5] Yang Z, Li N, He Y, Meng J, Wang J. Genetic Characterization of DH13M98, *Umatilla Virus*, Isolated from *Culex tritaeniorhynchus Giles* in Yunnan Province, China. Vector Borne Zoonotic Dis. 2023;23:35–43. 10.1089/vbz.2022.0031 36595376

[6] Santos PD, Ziegler U, Szillat KP, Szentiks CA, Strobel B, Skuballa J, et al. In action-an early warning system for the detection of unexpected or novel pathogens. Virus Evol. 2021;7:veab085. 10.1093/ve/veab085 34703624 PMC8542707

[7] Maan S, Maan NS, Samuel AR, Rao S, Attoui H, Mertens PPC. Analysis and phylogenetic comparisons of full-length VP2 genes of the 24 bluetongue virus serotypes. J Gen Virol. 2007;88:621–30. 10.1099/vir.0.82456-0 17251581

[8] Huismans H, Erasmus BJ. Identification of the serotype-specific and group-specific antigens of bluetongue virus. Onderstepoort J Vet Res. 1981;48:51–8. 6273773

[9] Mertens PP, Pedley S, Cowley J, Burroughs JN, Corteyn AH, Jeggo MH, et al. Analysis of the roles of bluetongue virus outer capsid proteins VP2 and VP5 in determination of virus serotype. Virology. 1989;170:561–5. 10.1016/0042-6822(89)90447-9 2543130

[10] Jesse ST, Ciurkiewicz M, Siesenop U, Spitzbarth I, Osterhaus ADME, Baumgärtner W, et al. Molecular characterization of a bovine adenovirus type 7 (Bovine Atadenovirus F) strain isolated from a systemically infected calf in Germany. Virol J. 2022;19:89. 10.1186/s12985-022-01817-y 35610654 PMC9131638

[11] Kalantar KL, Carvalho T, de Bourcy CFA, Dimitrov B, Dingle G, Egger R, et al. IDseq-An open source cloud-based pipeline and analysis service for metagenomic pathogen detection and monitoring. Gigascience. 2020;9:giaa111. 10.1093/gigascience/giaa111 33057676 PMC7566497

[12] Tamura K, Stecher G, Kumar S. MEGA11: Molecular Evolutionary Genetics Analysis Version 11. Mol Biol Evol. 2021;38:3022–7. 10.1093/molbev/msab120 33892491 PMC8233496

[13] Polampalli S, Choughule A, Prabhash K, Amare P, Baisane C, Kabre S, et al. Role of RT-PCR and FISH in diagnosis and monitoring of acute promyelocytic leukemia. Indian J Cancer. 2011;48:60–7. 10.4103/0019-509X.75831 21248444

[14] Vermeulen J, De Preter K, Lefever S, Nuytens J, De Vloed F, Derveaux S, et al. Measurable impact of RNA quality on gene expression results from quantitative PCR. Nucleic Acids Res. 2011;39:e63. 10.1093/nar/gkr065 21317187 PMC3089491

[15] Weber F, Wagner V, Rasmussen SB, Hartmann R, Paludan SR. Double-stranded RNA is produced by positive-strand RNA viruses and DNA viruses but not in detectable amounts by negative-strand RNA viruses. J Virol. 2006;80:5059–64. 10.1128/JVI.80.10.5059-5064.2006 16641297 PMC1472073

