# Supplementary material for: Umatilla Virus in Zoo-Dwelling Cape Penguins with Hepatitis, Germany
Source: Emerg Infect Dis. 2024 Dec;30(12):2643–6. doi: 10.3201/eid3012.240498 (PMC11616631; doi:10.3201/eid3012.240498)
Supplement: Appendix 1 — Additional information for Umatilla virus in zoo-dwelling Cape penguins with hepatitis, Germany [file 24-0498-Techapp-s1.pdf]

Article DOI: https://doi.org/10.3201/eid3012.240498

EID cannot ensure accessibility for supplementary materials supplied by authors. Readers who have difficulty accessing supplementary content should contact the authors for assistance.

Umatilla Virus in Zoo-Dwelling Cape Penguins with Hepatitis, Germany

Appendix 1

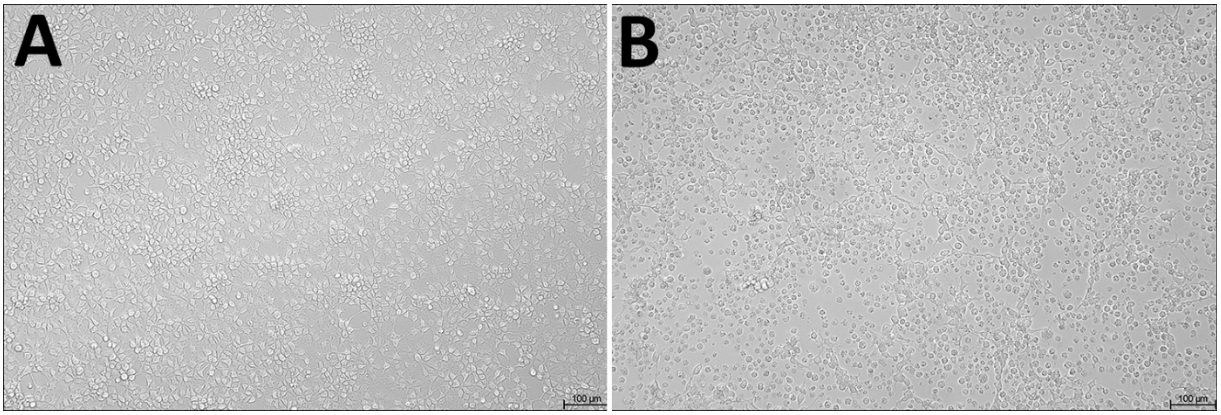

Appendix 1 Figure 1. Cytopathic effects (CPE) induced by Umatilla virus (UMAV) infection of chicken liver-derived cell line (LMH) cells at 3 days postinfection. (A) No CPE is observed in mock-infected LMH cells. (B) Extensive CPE characterized by cell rounding, aggregation, and detachment are visible in UMAV-infected LMH cells, including cell clumping and death. Photomicrographs were taken on a Leica DMi8 microscope. Scale bars, 100 µm.

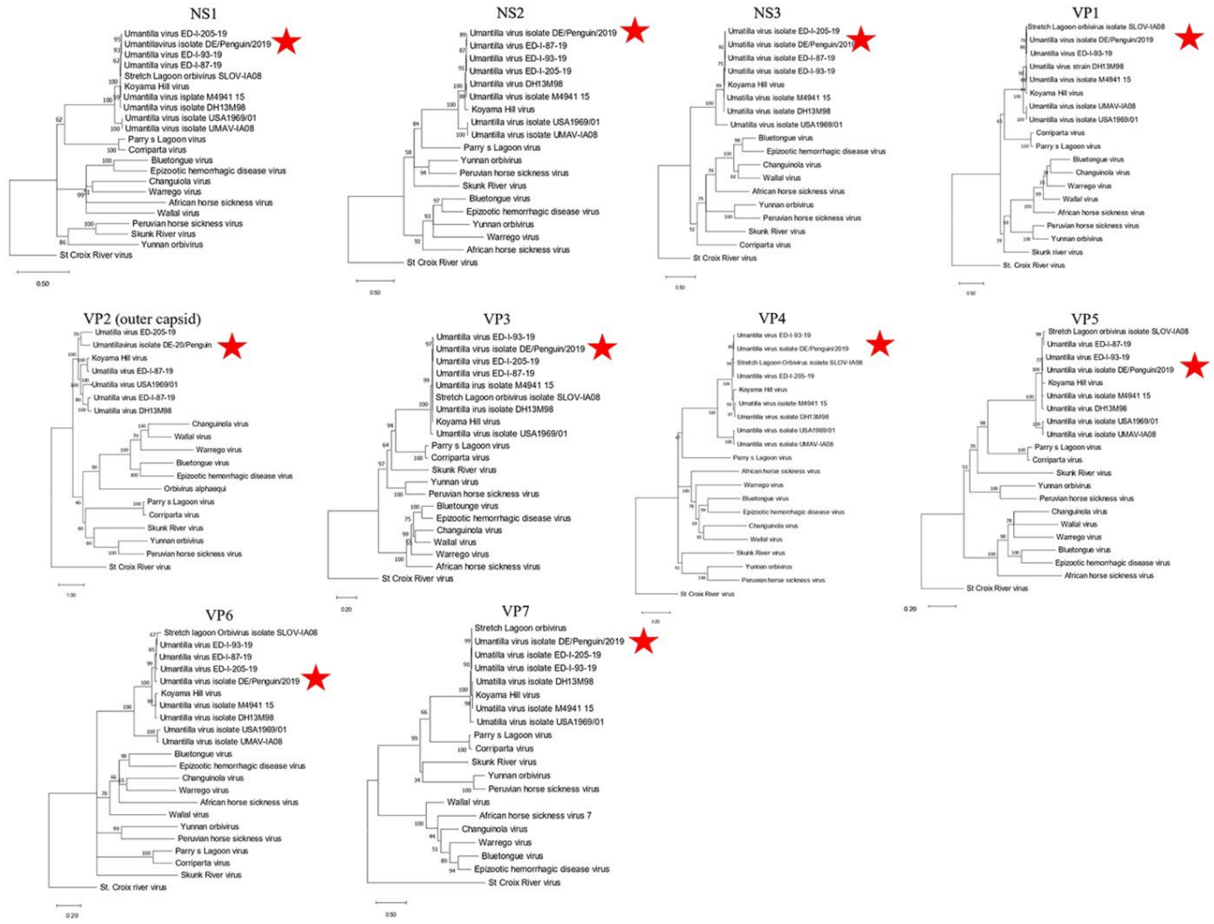

Appendix 1 Figure 2. Maximum likelihood (ML) phylogenetic trees of Umatilla virus (UMAV) and other Orbiviruses using protein amino acid sequences from 10 segments. St. Croix River virus was used as an outgroup. Red stars indicate the UMAV isolate DE/Penguin/2019 detected in this study. The ML tree was calculated with LG+G+I+F as substitution model with 1,000 bootstraps. Scale bar indicates the amino acid substitutions per site. All sequences used in the phylogenetic trees are listed with their GenBank accession number and names in Appendix 2 Table 2.

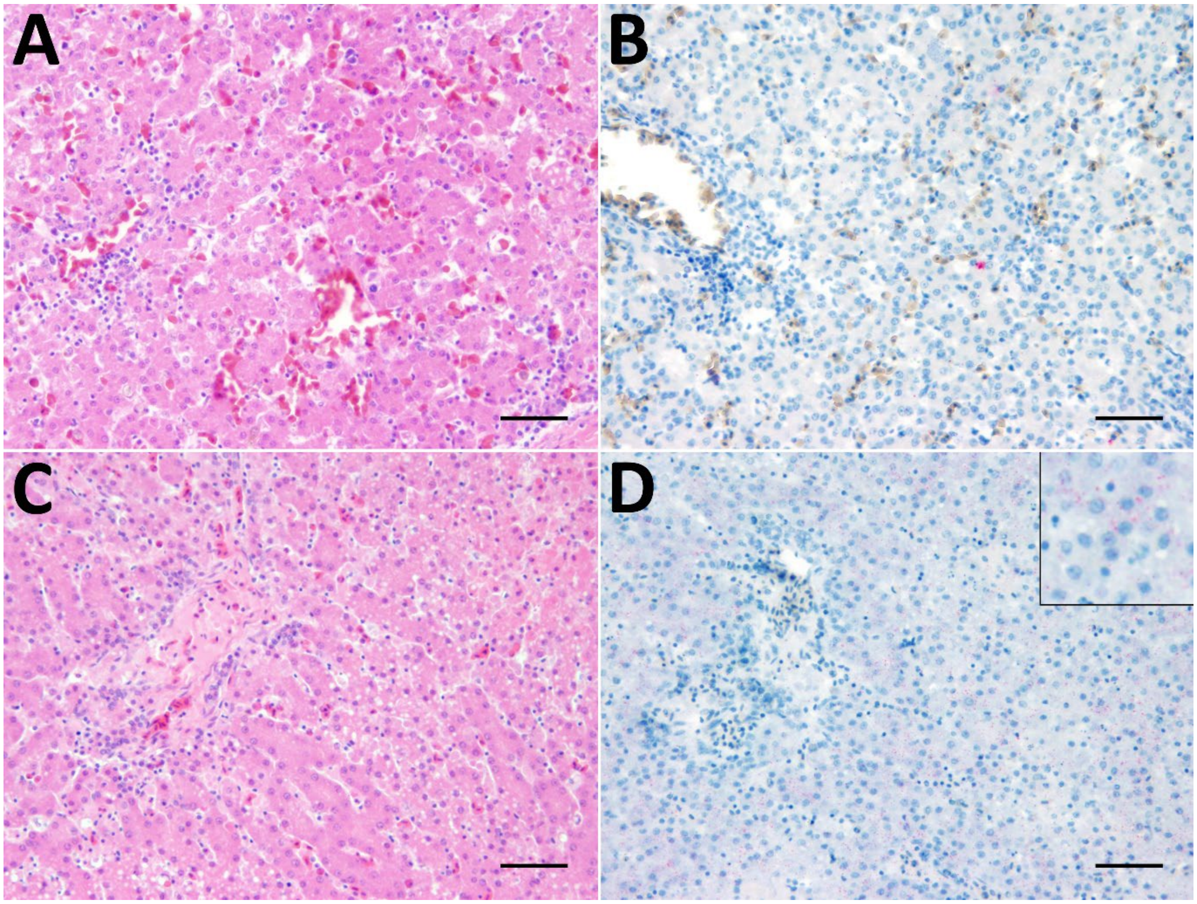

Appendix 1 Figure 3. Histopathologic findings and fluorescence in situ hybridization (FISH) results in archived formalin-fixed, paraffin-embedded tissue of 2 Umatilla virus (UMAV)-infected penguins during 2005–2011. (A) Hematoxylin and eosin (HE), liver of an UMAV-infected penguin from 2011. Moderate, multifocal to coalescing, hepatocellular necrosis. (B) FISH, liver (same 2011 penguin). UMAV RNA was demonstrated cytoplasmically in hepatocytes. (C) HE, liver of an UMAV-infected penguin from 2005. Mild, multifocal, hepatocellular necrosis. (D) FISH, liver (same 2005 penguin). Positive signals for UMAV RNA were located intracytoplasmically in hepatocytes, randomly distributed throughout the whole section. Inset: Multifocal, UMAV RNA-positive hepatocytes at higher magnification. Scale bars, 50 µm.
